# Diosgenin Alleviates Age‐Related Sarcopenia by Promoting Satellite Cell Proliferation and Myogenic Differentiation via Activation of the SIRT1/PGC‐1α Signaling Pathway

**DOI:** 10.1111/acel.70651

**Published:** 2026-07-30

**Authors:** Xin Zeng, Han Ding, Ziye Li, Qian Wang, Peiyao Guan, Tingting Wang, Jiayun Wang, Yansong Fu, Lizhang Chen, Hong Qin

**Affiliations:** ^1^ Department of Nutrition Science and Food Hygiene, Xiangya School of Public Health Central South University Changsha China; ^2^ Department of Epidemiology and Health Statistics, Xiangya School of Public Health Central South University Changsha China; ^3^ Hunan Provincial Key Laboratory of Clinical Epidemiology, Xiangya School of Public Health Central South University Changsha China; ^4^ College of Life Science and Medicine Zhejiang Science and Technology University Hangzhou China

**Keywords:** aging, diosgenin, sarcopenia, satellite cells, SIRT1/PGC‐1α

## Abstract

Age‐related sarcopenia is characterized by a progressive decline in skeletal muscle mass and function, with satellite cell dysfunction representing a central pathogenic mechanism. Diosgenin, a steroidal saponin derived from plants of the *Dioscorea* genus, has demonstrated potential anti‐aging properties; however, its role in sarcopenia remains unclear. In this study, naturally aged C57BL/6J mice and a D‐galactose (D‐gal)–induced senescent C2C12 cell model were employed to systematically investigate the effects of diosgenin on muscle function, satellite cell dynamics, and the sirtuin 1 (SIRT1)/peroxisome proliferator‐activated receptor gamma coactivator‐1 alpha (PGC‐1α) signaling pathway. Diosgenin treatment significantly improved forelimb grip strength and exercise endurance, increased the gastrocnemius muscle index, and enlarged muscle fiber cross‐sectional area in aged mice. Mechanistically, diosgenin upregulated the expression of myokines meteorin‐like protein (METRNL) and insulin‐like growth factor 1 (IGF‐1) at both mRNA and protein levels, increased the number of proliferative satellite cells positive for paired box 7 (Pax7) and Ki67, and enhanced the expression of myogenic markers, including myogenic factor 5 (Myf5), Pax7, and myosin heavy chain II (MyHC II). These effects were mediated by direct activation of SIRT1, leading to deacetylation of PGC‐1α. Notably, pharmacological inhibition of SIRT1 with EX527 markedly abrogated the diosgenin‐induced effects. Molecular docking and cellular thermal shift assays further confirmed the direct interaction between diosgenin and SIRT1. Collectively, these findings demonstrate that diosgenin alleviates age‐related sarcopenia by activating the SIRT1/PGC‐1α signaling pathway to promote satellite cell proliferation and myogenic differentiation, highlighting its potential as a promising therapeutic candidate for sarcopenia.

## Graphical abstract

As a critical bioactive component of *Polygonatum sibiricum*, diosgenin improves age‐related declines in skeletal muscle mass and function by activating the SIRT1/PGC‐1α pathway, upregulating the expression of the myokine METRNL and IGF‐1, and promoting satellite cell proliferation and myogenic differentiation.

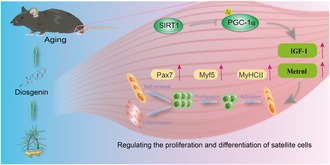

## 1 Introduction

Age‐related sarcopenia is a syndrome closely associated with aging and is characterized by a progressive decline in skeletal muscle mass and function. It is primarily manifested by reduced muscle strength, impaired physical performance, and an increased risk of falls, which severely compromises quality of life in older adults and elevates all‐cause mortality (Cruz‐Jentoft and Sayer 2019; Petermann‐Rocha et al. 2022; Shafiee et al. 2017). With the rapid acceleration of global population aging, sarcopenia has emerged as an increasingly serious public health concern (Anker et al. 2016; Dent et al. 2019). Currently, the main clinical interventions for sarcopenia include resistance exercise and hormone replacement therapy; however, their effectiveness remains suboptimal due to limitations in applicability, safety, and long‐term adherence (Beckwee et al. 2019; Cruz‐Jentoft et al. 2019). Therefore, identifying effective, safe, and easily implementable strategies for the prevention and treatment of sarcopenia holds substantial scientific and societal significance.

The onset and progression of sarcopenia are closely associated with impaired skeletal muscle regeneration, among which satellite cell dysfunction represents a central pathogenic mechanism (Sousa‐Victor et al. 2022; Verdijk et al. 2014). Satellite cells, as resident adult stem cells in skeletal muscle tissue, play a crucial role in muscle growth, repair, and regeneration (Dumont et al. 2015; Yin et al. 2013). During aging, both the number of satellite cells and their capacities for self‐renewal, proliferation, and differentiation are markedly reduced, leading to disrupted muscle fiber homeostasis and compromised regenerative potential, ultimately resulting in declines in muscle mass and function (Kolonay et al. 2024; Sahinyan et al. 2023). Consequently, targeting satellite cells to enhance their proliferative and myogenic capacities is considered a key strategy for reversing the progression of age‐related sarcopenia.

PGC‐1α plays a pivotal role in regulating satellite cell function, and its activity is tightly controlled by post‐translational acetylation modifications (Gurd 2011; Suntar et al. 2020). Upon deacetylation and activation, PGC‐1α indirectly regulates muscle homeostasis by modulating myokine signaling. Specifically, PGC‐1α upregulates the expression and secretion of key myokines such as METRNL and IGF‐1, thereby governing satellite cell fate, promoting their transition from quiescence to proliferation, and enhancing their differentiation toward the myogenic lineage. Through these mechanisms, PGC‐1α delays cellular senescence and plays an essential role in maintaining skeletal muscle homeostasis (Guo, Zhang, et al. 2023; Lee et al. 2020; Rao et al. 2014; Schiaffino and Mammucari 2011). SIRT1, a nicotinamide adenine dinucleotide (NAD^+^)‐dependent deacetylase, activates its critical downstream coactivator PGC‐1α through deacetylation (Kim et al. 2022). During aging, a decline in intracellular NAD^+^ levels leads to reduced SIRT1 activity, resulting in impaired PGC‐1α function, disruption of satellite cell homeostasis, and compromised muscle regenerative capacity, which together constitute an important molecular basis for the development of sarcopenia (Yang et al. 2024; Zhu et al. 2023). Therefore, enhancing satellite cell proliferation and differentiation by targeting the SIRT1/PGC‐1α axis is regarded as a promising strategy for combating sarcopenia.

Accumulating pharmacological evidence indicates that *Polygonatum* species and their bioactive constituents exhibit significant potential in anti‐aging, fatigue alleviation, and physical performance enhancement (Cui et al. 2018; Zhao et al. 2018). Diosgenin is a steroidal saponin monomer extracted from plants of the *Dioscoreaceae* family and represents one of the key bioactive components of the traditional Chinese medicinal herb *Polygonatum* (Chen et al. 2024). As a major active constituent of *Polygonatum*, diosgenin has been shown to activate the SIRT1 signaling pathway and effectively alleviate metabolic disorders in hepatic disease models (Vijayakumar et al. 2010). Recent studies have further demonstrated that diosgenin improves dysregulated glucose and lipid metabolism via SIRT1‐dependent mechanisms and exerts protective effects in multiple aging‐related pathological models (Q. Yin et al. 2025; Yuan et al. 2023). However, whether diosgenin can directly target SIRT1 signaling in muscle satellite cells to ameliorate age‐related sarcopenia remains unclear. Given the central role of the SIRT1/PGC‐1α axis in regulating muscle function, the present study employed naturally aged mouse models and a D‐gal–induced senescent C2C12 cell model to systematically evaluate the effects of diosgenin on skeletal muscle mass and function, satellite cell proliferation and differentiation, and the SIRT1/PGC‐1α signaling pathway, with the aim of identifying a novel candidate compound and mechanistic basis for the pharmacological intervention of age‐related sarcopenia.

## 2 Materials and Methods

### 2.1 Chemicals and Reagents

Diosgenin (Cat. #HY‐N0134) and SIRT1 inhibitor EX‐527 (Cat. #HY‐15452) were purchased from MedChemExpress (Monmouth Junction, USA). D‐gal (Cat. #G6223) and sodium carboxymethyl cellulose (CMC‐Na; Cat. #C104977) were obtained from Macklin (Shanghai, China). Mouse METRNL enzyme‐linked immunosorbent assay (ELISA) kit (Cat. #JL230125) and mouse IGF‐1 ELISA kit (Cat. #JL230228) were obtained from JONLNBIO (Shanghai, China). Senescence‐associated β‐galactosidase (SA‐β‐gal) staining kit was purchased from Solarbio (Beijing, China). TRIzol reagent, a first‐strand cDNA synthesis kit for quantitative polymerase chain reaction (qPCR), and a SYBR Green–based qPCR Master Mix were purchased from TransGen Biotech (Beijing, China). Antibodies against PGC‐1α (Cat. #AP20542C), Pax7 (Cat. #A7335), Myf5 (Cat. #A116227), p53 (Cat. #A21630), p21 (Cat. #A1483), MyHC II (Cat. #A15293), and GAPDH (Cat. #AC033) were purchased from ABclonal (Wuhan, China). Antibody against SIRT1 (Cat. #DF6033) was obtained from Affinity Biosciences (Liyang, China). Pan‐acetyl‐lysine antibody (Cat. #A0671) was purchased from Proteintech (Wuhan, China). Antibodies against p16 (Cat. #GB111143) and Ki67 (Cat. #GB111141) were purchased from Servicebio (Wuhan, China). *Polygonatum sibiricum* polysaccharide (PSP) was obtained from Yuanye Bio‐Technology (Shanghai, China). Isoflurane was purchased from RWD Life Science (Shenzhen, China). Phosphate‐buffered saline (PBS), 4% paraformaldehyde, xylene, anhydrous ethanol, graded ethanol, limonene‐based clearing agent, neutral resin, hematoxylin, eosin, EDTA buffer (pH 8.0), 3% hydrogen peroxide (H_2_O_2_), bovine serum albumin (BSA), DAB chromogen solution, 4′,6‐diamidino‐2‐phenylindole (DAPI), optimal cutting temperature (OCT) compound, radioimmunoprecipitation assay (RIPA) buffer, Tris‐buffered saline with Tween 20 (TBST), sodium dodecyl sulfate (SDS) loading buffer, and Protein A/G magnetic beads were obtained from Servicebio (Wuhan, China). CCK‐8 kit was purchased from AbMole (Shanghai, China). Bicinchoninic acid (BCA) protein assay kit and enhanced chemiluminescence (ECL) kit were obtained from GlpBio (Montclair, USA). Nitrocellulose (NC) membranes were purchased from Cytiva (Marlborough, USA). Skim milk was obtained from BD Biosciences (San Jose, USA). Fluorescent secondary antibodies (Alexa Fluor 488‐conjugated and Alexa Fluor 568‐conjugated) were purchased from Thermo Fisher Scientific (Waltham, USA). Protease and deacetylase inhibitors were obtained from Roche (Basel, Switzerland). All other chemicals and reagents were of analytical grade and commercially available.

### 2.2 Animals and Interventions

Specific pathogen–free (SPF) male C57BL/6J mice were used in this study and obtained from Slack Jinda Experimental Animal Co. Ltd. (Changsha, China). A young control group consisted of 2‐month‐old mice (*n* = 9), while 18‐month‐old mice were used to establish the naturally aged model (*n* = 36). The 18‐month‐old mice were randomly assigned to four groups (*n* = 9 per group): aging control group, low‐dose diosgenin group (DL; 30 mg/kg body weight), high‐dose diosgenin group (DH; 90 mg/kg body weight), and positive control group treated with PSP (200 mg/kg body weight). Diosgenin and PSP were dissolved in 0.5% sodium carboxymethyl cellulose (CMC‐Na) solution and administered once daily by oral gavage for 8 consecutive weeks. Diosgenin was freshly prepared in 0.5% CMC‐Na at concentrations of 2.1 and 6.3 mg/mL for the low‐ and high‐dose groups, respectively. PSP was dissolved in 0.5% CMC‐Na at a concentration of 80 mg/mL. The young control (YC) group and old control (OC) group received an equal volume of 0.5% CMC‐Na solution. Throughout the experimental period, animals were housed under standard conditions with free access to food and water, and all efforts were made to minimize animal suffering and distress. Animal health status was monitored daily, and humane endpoints were applied when necessary. Randomization was performed prior to treatment allocation. Investigators involved in behavioral assessments, histological analysis, and data quantification were blinded to group assignments throughout the study. All animal experimental procedures were conducted in strict accordance with the ARRIVE guidelines and the ethical standards for laboratory animal welfare in China, and were approved by the Animal Welfare and Ethics Committee of Central South University (approval number: CSU‐2024‐0182; date: June 27, 2024).

### 2.3 Grip Strength Test

Forelimb grip strength was assessed using the Kondziela inverted screen test. Mice were placed on a metal wire grid, which was slowly inverted, and the latency to fall was recorded. Each mouse was tested five times with an intertrial interval of at least 30 s, and the average latency was calculated as the final grip strength value.

### 2.4 Endurance Test

A lead weight equivalent to 5% of the mouse's body weight was attached to the tail, and the mice were placed in a swimming tank with the water temperature maintained at 25°C ± 1°C. The mice were allowed to swim until exhaustion, which was defined as the inability to keep the head above water for 10 consecutive seconds. The time from immersion to exhaustion was recorded as the endurance swimming time.

### 2.5 Tissue Collection and Processing

24 h after the final administration, mice were fasted for 12 h with free access to water and then anesthetized by inhalation of 3% isoflurane. Whole blood was collected from the retro‐orbital venous plexus and allowed to stand at room temperature for 30 min, followed by centrifugation at 3000 rpm for 15 min at 4°C to obtain serum. Serum samples were aliquoted and stored at −80°C for subsequent analyses. After blood collection, mice were euthanized by rapid cervical dislocation. Bilateral gastrocnemius muscles were carefully excised and weighed using a precision electronic balance. The gastrocnemius muscle index was calculated as the wet weight of the gastrocnemius muscle (mg) divided by body weight (g) × 100%. The left gastrocnemius muscle was immediately fixed in 4% paraformaldehyde in PBS for 24 h for histological analysis. The right gastrocnemius muscle was sectioned, rapidly frozen in liquid nitrogen, and subsequently stored at −80°C until further use. For histopathological analysis, the left gastrocnemius muscle was fixed in 4% paraformaldehyde for 24 h, then dehydrated through a graded ethanol series, cleared in xylene, and embedded in paraffin. Paraffin‐embedded tissues were sectioned into continuous 4‐μm‐thick slices for subsequent H&E and IHC staining.

### 2.6 Hematoxylin and Eosin (H&E) Staining and Cross‐Sectional Area Measurement of Skeletal Muscle

Sections were stained with hematoxylin for 4 min, differentiated in 0.8% acid alcohol for 2 s, and rinsed under running water for bluing. Subsequently, sections were counterstained with eosin for 20 s, briefly differentiated in 95% ethanol for 5 s, and dehydrated twice in absolute ethanol for 2 min each. The sections were cleared in a limonene‐based clearing agent for 10 min and mounted with neutral resin. H&E staining was used to evaluate skeletal muscle histopathological morphology. Images were captured using an EVOS M7000 bright‐field imaging system (Thermo Fisher Scientific, Waltham, USA) equipped with infinite correction optics, a 60 mm long working distance condenser, and a 3.2 MP monochrome CMOS camera. All images were acquired under adjustable LED illumination using a (20×/40×) objective. The cross‐sectional area of muscle fibers was quantified using Image‐Pro Plus software by randomly measuring at least 200 muscle fibers per sample.

### 2.7 Immunohistochemical (IHC) Staining

Paraffin sections were subjected to heat‐induced antigen retrieval in pH 8.0 ethylenediaminetetraacetic acid (EDTA) buffer for 30 min. After cooling to room temperature, sections were washed with PBS. Endogenous peroxidase activity was quenched by incubating the sections with 3% H_2_O_2_ at room temperature in the dark for 25 min, followed by PBS washing. Sections were then blocked with 3% bovine serum albumin (BSA) at room temperature for 30 min to prevent nonspecific binding. After removal of the blocking solution, sections were incubated overnight at 4°C in a humidified chamber with a primary antibody against p16 (1:500; Servicebio, Wuhan, China; Cat. #GB111143). After washing with PBS, sections were incubated with a horseradish peroxidase (HRP)–conjugated goat anti‐rabbit IgG secondary antibody (1:200; Servicebio, Wuhan, China; Cat. #GB23303) at room temperature for 50 min. Following three washes with PBS, immunoreactivity was visualized using freshly prepared 3,3′‐diaminobenzidine (DAB) working solution, with color development monitored under an EVOS M7000 bright‐field imaging system. Nuclei were counterstained with hematoxylin for 3 min, followed by bluing under running water, dehydration through graded ethanol, clearing in xylene, and mounting with neutral resin. In this study, both positive and negative controls were included to ensure the reliability of the staining results. The positive control showed the expected positive signal, and the negative control showed no specific staining, indicating that the experimental system was valid. Stained sections were examined under a light microscope, and brown‐yellow signals were considered positive immunostaining. For IHC quantification, one tissue section per group was examined. From this single section, three non‐overlapping fields were randomly selected at 400× magnification. The extent of positive staining (percentage of positive area) was analyzed using ImageJ software for each field, and each field was treated as an individual data point. Statistical comparisons between groups were performed based on these three fields per group. Representative images were captured for further analysis.

### 2.8 Immunofluorescence (IF) Staining

Skeletal muscle tissues were fixed in 4% paraformaldehyde, dehydrated, embedded in OCT compound, and rapidly frozen in liquid nitrogen. Frozen sections were cut at a thickness of 10 μm using a cryostat. After antigen retrieval and blocking, sections were incubated simultaneously with primary antibodies against Pax7 and Ki67 at 4°C overnight. Pax7 is a hallmark transcription factor of satellite cells, maintaining their stem cell properties and self‐renewal ability, while Ki67 is a nuclear protein expressed in all actively proliferating cells except those in the quiescent phase, widely used as a marker of cell proliferation. On the following day, sections were incubated with the corresponding fluorescent secondary antibodies, and nuclei were counterstained with DAPI. The primary antibodies used were: anti‐Pax7 (1:1000; Servicebio, Wuhan, China; Cat. #GB113190) and anti‐Ki67 (1:300; Servicebio, Wuhan, China; Cat. #GB121141). The secondary antibodies were: CY3‐conjugated goat anti‐rabbit IgG (1:300; Servicebio, Wuhan, China; Cat. #GB21303) for Pax7 detection and Alexa Fluor 488‐conjugated goat anti‐mouse IgG (1:400; Servicebio, Wuhan, China; Cat. #GB25301) for Ki67 detection. Images were acquired using an EVOS M7000 inverted fluorescence imaging system. For quantification, one tissue section per group was examined. From this single section, three non‐overlapping fields were randomly selected at 400× magnification. The number of double‐positive (Pax7^+^/Ki67^+^) satellite cells was manually counted for each field, and each field was treated as an individual data point. Statistical comparisons between groups were performed based on these three fields per group. Representative images were captured to illustrate the findings.

### 2.9 Cell Culture and Induction of Differentiation

C2C12 cells were obtained from the Advanced Research Center of Central South University (Changsha, China) and cultured in high‐glucose Dulbecco's Modified Eagle Medium (DMEM; Biochannel, Nanjing, China) supplemented with 10% fetal bovine serum (FBS; Gibco, Grand Island, USA), 100 U/mL penicillin, and 100 μg/mL streptomycin (Servicebio, Wuhan, China) at 37°C in a humidified incubator with 5% CO_2_. When cells reached 80%–90% confluence, the growth medium was replaced with differentiation medium containing 2% horse serum (Gibco, Grand Island, USA) to induce myogenic differentiation. The differentiation medium was refreshed every 24 h for a total of 5 days.

### 2.10 Cell Viability and Proliferation Assay

To evaluate the potential cytotoxicity of diosgenin on differentiated myotubes and its pro‐proliferative effect on undifferentiated myoblasts, cell viability was assessed using the cell counting kit‐8 (CCK‐8; AbMole, Shanghai, China) assay.

Cytotoxicity assay in myotubes: C2C12 cells were seeded in 96‐well plates at a density of 7 × 10^3^ cells per well in 100 μL of growth medium and cultured overnight. When cells reached 80%–90% confluence, the growth medium was replaced with differentiation medium containing 2% horse serum to induce myogenic differentiation. On Day 3 of differentiation, cells were treated with D‐gal for 24 h, followed by diosgenin (0.5, 1, and 2 μM) or EX527 for another 24 h. Cells were then harvested on Day 5, and cell viability was measured using the CCK‐8 assay. This experiment was performed to assess the cytotoxicity of diosgenin on myotubes and to determine a safe concentration range.

Proliferation assay in myoblasts: Based on the non‐cytotoxic concentration range (0.5–2 μM) identified above, the following groups were designed to evaluate the pro‐proliferative effect of diosgenin on myoblasts: blank control group (complete DMEM), aging model group (100 μM D‐gal), diosgenin intervention groups (0.5, 1, and 2 μM diosgenin + 100 μM D‐gal), and PSP group (400 μg/mL PSP + 100 μM D‐gal). Cells were treated for 24 h, after which 10 μL of CCK‐8 solution was added to each well and incubated at 37°C for 2 h. Absorbance at OD450 was measured using a microplate reader (Thermo Fisher Scientific, Waltham, USA). The cell proliferation inhibition rate was calculated using the following formula: Inhibition rate (%) = [1 − (OD_experimental group_ − OD_blank group_)/(OD_control group_ − OD_blank group_)] × 100%.

### 2.11 β‐Gal Staining

Cellular senescence of C2C12 cells was assessed using a SA‐β‐gal staining kit according to the manufacturer's instructions. C2C12 cells were seeded in six‐well plates and cultured overnight at 37°C in a 5% CO_2_ incubator. After the corresponding treatments, the culture medium was removed, and cells were washed once with PBS. Cells were then fixed with 1 mL of β‐gal fixation solution at room temperature for 15 min. Following fixation, cells were washed three times with PBS. After removing PBS, 1 mL of staining working solution was added to each well. Plates were incubated overnight at 37°C in a sealed 6‐well plate to prevent evaporation. Finally, images were captured using an EVOS M7000 imaging system. For SA‐β‐gal quantification, due to the high cell density after differentiation, it was not feasible to count all cells in each well. Therefore, for each well, one randomly selected non‐overlapping field was captured at 400× magnification. The percentage of SA‐β‐gal positive cells was calculated as (number of blue‐stained cells/total number of cells in that field) × 100%. Each well provided one data point, and three independent wells per group were used as three replicates. Quantification was performed using ImageJ software.

### 2.12 qPCR

Total RNA was extracted from treated mouse skeletal muscle tissues and C2C12 cells using TRIzol reagent. RNA concentration and purity were measured using a NanoDrop spectrophotometer (Thermo Fisher Scientific, Waltham, USA), with an A_260_/A_280_ ratio of 1.8–2.0 considered acceptable. One microgram of total RNA was reverse‐transcribed into cDNA using a first‐strand cDNA synthesis kit. PCR was performed using SYBR Green qPCR mix on a real‐time PCR system (Roche, Basel, Switzerland). The amplification program was set as follows: pre‐denaturation at 95°C for 30 s; 40 cycles of 95°C for 5 s and 60°C for 30 s for annealing/extension; followed by melting curve analysis. GAPDH was used as the internal reference gene. Primer sequences for all target genes were designed and synthesized by Servicebio (Wuhan, China). Gene expression levels were calculated using the 2^−ΔΔCt^ method. Target genes analyzed included *Metrnl*, *Igf1*, *Cpt1b* (CPT‐1β is a downstream target of PGC‐1α), and *Gapdh*.

The primer sequences are as follows: (3′–5′): *Metrnl*, forward‐TGACTTTGTTGTCCGAGGCTT, reverse‐CCCTGGTCGTACTCCACACT; *Igf1*, forward‐GTGGATGCTCTTCAGTTCGTGT, reverse‐CTTTCCTTCTCCTTTGCAGCTT; *Cpt1b*, forward‐TTAGGGGTGTGTACCCTGGC, reverse‐CTCCGGTGGAGAAGATGACC; *Gapdh*, forward‐CCTCGTCCCGTAGACAAAATG, reverse‐TGAGGTCAATGAAGGGGTCGT.

### 2.13 ELISA

Protein levels of METRNL and IGF‐1 in serum, skeletal muscle tissue homogenates, and C2C12 cell culture supernatants were measured using commercial ELISA kits according to the manufacturer's instructions. For tissue homogenate preparation, skeletal muscle tissues were minced and homogenized in ice‐cold PBS (1:9 w/v) using a tissue homogenizer, then centrifuged at 12,000 rpm for 15 min at 4°C. The supernatants were collected for ELISA analysis. Standards and samples were added to pre‐coated 96‐well plates and incubated at room temperature for 2 h. After washing, biotin‐labeled detection antibodies were added and incubated at room temperature for 1 h. Plates were washed again, followed by incubation with HRP‐conjugated streptavidin at room temperature for 30 min. After a final wash, 3,3′,5,5′‐tetramethylbenzidine (TMB) substrate solution was added for color development at room temperature in the dark for 15–30 min, and the reaction was stopped. OD_450_ was measured using a microplate reader, and protein concentrations were calculated based on the standard curve.

### 2.14 Western Blot (WB)

Frozen skeletal muscle tissues or collected C2C12 cells were lysed in pre‐cooled RIPA buffer (GlpBio, Montclair, USA) using a tissue homogenizer or ultrasonic disruptor. Lysates were centrifuged at 12,000 rpm for 15 min at 4°C, and the supernatants were collected. Protein concentrations were determined using a BCA protein assay kit (GlpBio, Montclair, USA). Equal amounts of protein were separated by SDS–polyacrylamide gel electrophoresis (SDS‐PAGE) and transferred onto NC membranes (Cytiva, Marlborough, USA). Membranes were blocked with 5% skim milk at room temperature for 1 h and incubated with the following primary antibodies at 4°C overnight: SIRT1 (1:1000; Affinity Biosciences, Liyang, China; Cat. #DF6033), PGC‐1α (1:1000; ABclonal, Wuhan, China; Cat. #AP20542C), p53 (1:1000; ABclonal, Wuhan, China; Cat. #A21630), p21 (1:1000; ABclonal, Wuhan, China; Cat. #A1483), Myf5 (1:1000; ABclonal, Wuhan, China; Cat. #A116227), MyHC II (1:1000; ABclonal, Wuhan, China; Cat. #A15293), Pax7 (1:1000; ABclonal, Wuhan, China; Cat. #A7335), and GAPDH (1:20,000; ABclonal, Wuhan, China; Cat. #AC033). After washing with TBST, membranes were incubated with corresponding HRP‐conjugated secondary antibodies at room temperature for 1 h. Signals were developed using an ECL kit (GlpBio, Montclair, USA) and captured with a chemiluminescence imaging system (Azure Biosystems, Dublin, USA). Band intensities were quantified using ImageJ software, and target protein expression levels were normalized to GAPDH.

### 2.15 Immunoprecipitation (IP) and Acetylation Assay

C2C12 cells and tissue homogenates from different experimental groups were lysed in RIPA buffer containing protease and deacetylase inhibitors. The lysates were centrifuged at 12,000 rpm for 15 min at 4°C, and the supernatants were collected as protein samples. Protein concentrations were determined using a BCA kit. A total of 500 μg of protein from each sample was divided into two equal portions: one for the negative control (IgG) and one for the target antibody (PGC‐1α). Each portion was incubated with either 10 μg of rabbit IgG or 10 μg of PGC‐1α antibody together with 20 μL of Protein A/G magnetic beads at 4°C for 1 h to form antibody–bead complexes. The resulting antibody–bead complexes were then added back to the corresponding protein portions and incubated overnight at 4°C with rotation. The next day, the beads were washed five times with pre‐chilled lysis buffer. Proteins were eluted by adding 2× SDS loading buffer and boiling at 95°C for 10 min. Immunoprecipitates were analyzed by WB, with acetyl‐lysine antibody used to detect the acetylation level of PGC‐1α, and total PGC‐1α detected by PGC‐1α antibody as a loading control.

### 2.16 Molecular Docking

The crystal structure of human SIRT1 protein (PDB ID: 4ZZJ) was downloaded from the Protein Data Bank (PDB) (https://www.rcsb.org/). The original ligand and water molecules were removed using PyMOL software, and hydrogen atoms were added and Gasteiger charges calculated using AutoDock Tools. The 3D structure of diosgenin (CID: 99474) was obtained from the PubChem database (https://pubchem.ncbi.nlm.nih.gov/) and energy‐minimized using Chem3D software. Molecular docking was performed with AutoDock Vina, centering the docking grid on the known active site of SIRT1. Binding free energy ≤ −5.0 kcal/mol was considered indicative of potential binding activity. The docking pose and binding pocket were visualized and analyzed using PyMOL.

### 2.17 Cellular Thermal Shift Assay (CETSA)

The CETSA was performed to evaluate whether diosgenin directly binds to and stabilizes the SIRT1 protein. This method is based on the principle that ligand binding typically increases the thermal stability of a target protein, protecting it from heat‐induced aggregation and denaturation. C2C12 cells were seeded in six‐well plates at a density of 2 × 10^6^ cells/well. After cell attachment, cells were treated with 10 μM diosgenin for 2 h. Cells were collected, washed twice with prechilled PBS, and resuspended in lysis buffer containing protease inhibitors. Cells were lysed by sonication (20% power, 3 s on/3 s off, 10 cycles) and centrifuged at 12,000 rpm for 15 min at 4°C to obtain the cell lysate. The lysate was divided equally into six tubes and heated in a gradient of temperatures (37°C, 40°C, 45°C, 50°C, 55°C, and 60°C) for 3 min in a water bath, followed by immediate cooling on ice. The samples were centrifuged at 20,000 rpm for 20 min at room temperature, and the supernatants were collected. Equal amounts of supernatant were subjected to WB analysis to assess thermal stability changes of SIRT1 protein.

### 2.18 Statistical Analysis

All data are presented as mean ± standard error of the mean (SEM). Statistical analyses were performed using GraphPad Prism software. The Shapiro–Wilk test was first used to assess the normality of the data. For data that were normally distributed with equal variances, one‐way analysis of variance (ANOVA) was performed. If significant differences were detected among groups (*p*‐value < 0.05), Tukey's multiple comparisons test was used for pairwise comparisons. For data that were not normally distributed or had unequal variances, the nonparametric Kruskal–Wallis test was applied, followed by Dunn's multiple comparisons test if significant differences were observed. A *p*‐value < 0.05 was considered statistically significant.

## 3 Results

### 3.1 Diosgenin Improved Aging‐Related Phenotypes in Mice

**FIGURE 1:**
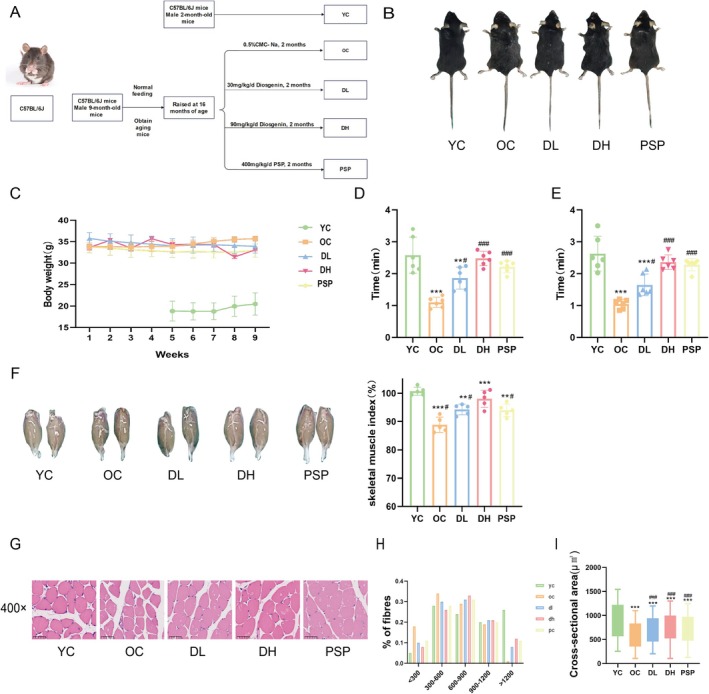
Diosgenin ameliorated age‐related declines in muscle function and mass in naturally aged mice. (A) Schematic diagram of the experimental design in mice. (B) Representative gross appearance of mice in each group. (C) Body weight changes of mice in each group during the experimental period. (D) Results of the hanging time test. (E) Results of the weighted swimming exhaustion time test. (F) Representative gross morphology of the gastrocnemius muscle and quantitative analysis of the gastrocnemius index (gastrocnemius wet weight/body weight × 100%). (G) Representative H&E‐stained images of gastrocnemius muscle from each group. Scale bar = 275 μm. (H) Frequency distribution of muscle fiber cross‐sectional area. The x‐axis represents cross‐sectional area ranges (μm^2^), and the y‐axis represents the percentage of muscle fibers within each range. (I) Quantitative analysis of the average muscle fiber cross‐sectional area, with at least 200 muscle fibers randomly measured per group. Data are presented as mean ± SEM. **p‐value < 0.01, ***p‐value < 0.001 versus the YC group; ^#^ *p*‐value < 0.05, ^###^ *p*‐value < 0.001 versus the OC group. Statistical analysis was performed using one‐way ANOVA followed by Tukey's multiple comparisons test. All experiments were performed with at least three independent replicates.

In this study, naturally aged mice were used to evaluate the effects of diosgenin on age‐related sarcopenia (Figure 1A). Compared with the YC group, OC mice exhibited typical aging phenotypes, including sparse, coarse hair and uneven, dull coat color (Figure 1B). Importantly, no significant differences in body weight were observed between any of the treatment groups and the OC group throughout the intervention period, indicating that neither diosgenin (low or high dose) nor PSP treatment affected body weight in aged mice (Figure 1C). Regarding muscle function, grip strength and endurance tests showed that hanging time and weighted swimming time in OC mice were significantly reduced compared with YC mice (*p*‐value < 0.05), indicating pronounced declines in muscle strength and endurance (Figure 1D,E). After 8 weeks of diosgenin intervention, both functional parameters were significantly prolonged in all treatment groups (*p*‐value < 0.05), with the DH group showing the most pronounced improvement. In terms of muscle mass, the gastrocnemius of OC mice was reduced, and the gastrocnemius muscle index was significantly lower than that of YC mice (*p*‐value < 0.05), suggesting age‐related muscle loss (Figure 1F). H&E staining revealed thinner muscle fibers in OC mice, indicating evident muscle fiber atrophy (Figure 1G). Following intervention, both the diosgenin (DL and DH) and PSP groups showed a significant increase in the gastrocnemius muscle index (*p*‐value < 0.05). Analysis of muscle fiber cross‐sectional area distribution showed that fibers in YC mice were mainly within the > 1200 μm^2^ range, while OC mice fibers were primarily distributed in the 300–900 μm^2^ range. After treatment, the fiber distribution in aged mice shifted toward larger sizes, with the DH and PSP groups showing patterns shifting toward that of the YC mice (Figure 1H). Concurrently, a significant increase in the mean cross‐sectional area was observed compared to the OC group (*p*‐value < 0.05), indicating a marked, yet partial, recovery of muscle fiber morphology, as the area in the treatment groups remained significantly lower than that of the YC mice (Figure 1I). These results indicated that diosgenin effectively alleviated the decline in muscle function and mass in aged mice, demonstrating its potential to improve age‐related sarcopenia.

### 3.2 Diosgenin Suppressed the Expression of Aging‐Related Molecular Markers

**FIGURE 2:**
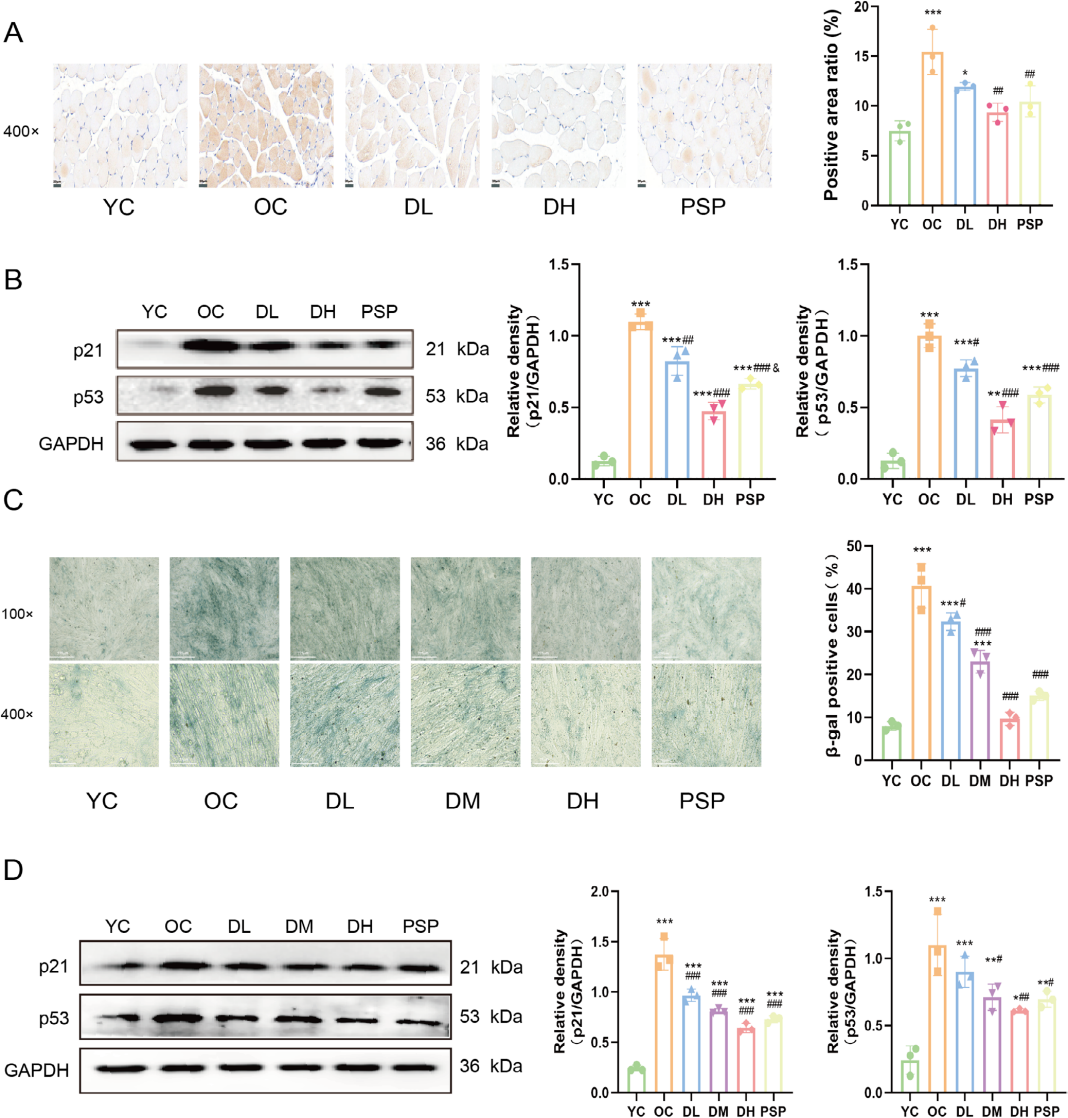
Diosgenin suppressed aging‐related molecular markers in vivo and in vitro. (A) Representative IHC staining images of p16 (brown‐yellow signal) in skeletal muscle tissues from each group of mice, with quantitative analysis. Scale bar = 75 μm. (B) Representative WB images and quantitative analysis of the aging‐related markers p53 and p21. (C) Representative SA‐β‐gal staining images of C2C12 cells at 100× and 400× magnification, with quantitative analysis. (D) Representative WB images and quantitative analysis of p53 and p21 protein levels in C2C12 cells. Data are presented as mean ± SEM. *p‐value < 0.05, **p‐value < 0.01, ***p‐value < 0.001 versus the YC group; ^#^ *p*‐value < 0.05, ^##^ *p*‐value < 0.01, ^###^ *p*‐value < 0.001 versus the OC group. ^&^ *p*‐value < 0.05 versus the PSP group. Statistical analysis was performed using one‐way ANOVA followed by Tukey's multiple comparisons test. All experiments were performed with at least three independent replicates.

Next, this study examined the effects of diosgenin on skeletal muscle aging. Compared with the YC group, the aging markers in the gastrocnemius muscle of OC mice were significantly elevated. IHC staining showed a notable increase in both the extent and intensity of p16‐positive signals, and WB analysis revealed that the protein expression levels of p53 and p21 were significantly upregulated (*p*‐value < 0.05). After diosgenin treatment, all dosage groups markedly alleviated these aging‐related changes: the extent and intensity of p16‐positive staining were significantly reduced, with the high‐dose group showing the greatest effect (*p*‐value < 0.05). Additionally, while p53 and p21 protein levels remained above the YC baseline, they exhibited a significant, dose‐dependent decrease compared to the OC group (*p*‐value < 0.05). The positive control PSP similarly suppressed p53 expression to a level comparable to the DH group, although DH was significantly more effective at reducing p21 (*p*‐value < 0.05) (Figure 2A,B).

A consistent pattern was also observed in the D‐gal–induced C2C12 senescence model. Compared with normal cells, the senescent model cells showed a significantly increased SA‐β‐gal positivity rate and upregulated p53 and p21 protein expression (*p*‐value < 0.05). Following intervention, both diosgenin (in a dose‐dependent manner) and PSP significantly reduced SA‐β‐gal‐positive cells and downregulated p53 and p21 expression (Figure 2C,D). These results showed that diosgenin exhibited anti‐senescence effects on skeletal muscle both in vivo and in vitro.

### 3.3 Diosgenin Promoted Proliferation and Myogenic Differentiation of Senescent Satellite Cells

**FIGURE 3:**
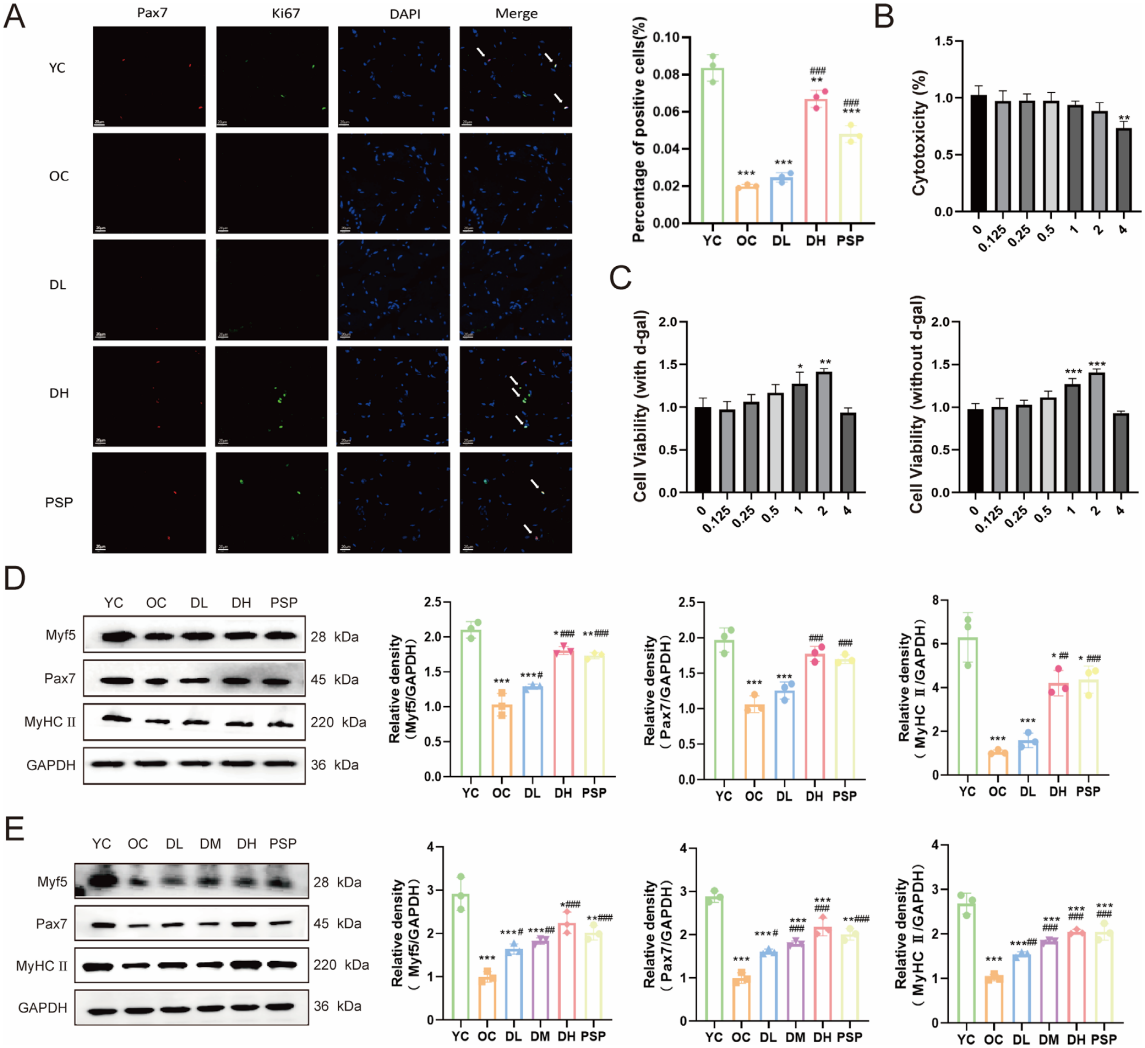
Diosgenin promoted proliferation and myogenic differentiation of senescent satellite cells. (A) Representative images of Pax7/Ki67 double IF staining in skeletal muscle tissues, with quantitative analysis. Green: Pax7 (satellite cell marker); red: Ki67 (proliferation marker); blue: DAPI (nuclei); yellow: Pax7^+^/Ki67^+^ double‐positive cells. Scale bar = 20 μm. (B) Cytotoxicity of diosgenin in differentiated myotubes (determination of safe concentration range, *n* = 3). (C) Proliferative activity of diosgenin in undifferentiated C2C12 myoblasts in the absence (left panel) or presence (right panel) of 100 μM D‐gal (*n* = 3). (D) Representative WB images and quantitative analysis of the myogenic differentiation markers Myf5, Pax7, and MyHC II in mouse skeletal muscle. GAPDH was used as the internal control. (E) Representative WB images and quantitative analysis of the myogenic differentiation markers Myf5, Pax7, and MyHC II in C2C12 cells. GAPDH was used as the internal control. Data are presented as mean ± SEM. *p‐value < 0.05, **p‐value < 0.01, ***p‐value < 0.001 versus the YC group; ^#^ *p*‐value < 0.05, ^##^ *p*‐value < 0.01, ^###^ *p*‐value < 0.001 versus the OC group. Statistical analysis was performed using one‐way ANOVA followed by Tukey's multiple comparisons test. All experiments were performed with at least three independent replicates.

Satellite cells, as adult stem cells in skeletal muscle, play a key role in maintaining muscle regeneration through their proliferative capacity. IF staining showed that, compared with the YC group, the proportion of Pax7^+^/Ki67^+^ proliferative cells in skeletal muscle of the OC group was significantly reduced (*p*‐value < 0.05), indicating that satellite cell self‐renewal was impaired during aging. After diosgenin treatment, the proportion of Pax7^+^/Ki67^+^ cells increased significantly, suggesting that quiescent satellite cells were activated and entered the proliferation phase. Among them, the DH group restored Pax7^+^/Ki67^+^ cell proportion closest to the YC group level (Figure 3A). A similar proliferation‐promoting effect was observed in the proliferation phase of C2C12 cells, and CCK‐8 results showed that cell number increased with diosgenin dose, reaching the optimal effect at 2 μM (*p*‐value < 0.05) (Figure 3B,C).

To evaluate the effect of diosgenin on myogenic differentiation, expression of key myogenic regulatory factors was assessed. WB results showed that, compared with the YC group, protein expression levels of the myogenic differentiation determinant Myf5, Pax7, and the mature myocyte marker MyHC II in skeletal muscle of the OC group were significantly decreased (*p*‐value < 0.05), indicating impaired myogenic differentiation during aging. Diosgenin treatment effectively upregulated the expression of these key factors (*p*‐value < 0.05), activated the myogenic transcriptional network, and promoted satellite cells to differentiate into mature myocytes. Notably, both the DH and positive control PSP groups showed robust improvements, restoring Myf5, Pax7, and MyHC II protein levels close to those of the YC group (*p*‐value > 0.05) (Figure 3D). In the D‐gal‐induced C2C12 senescence model, both diosgenin (in a dose‐dependent manner) and PSP treatment significantly increased the expression of these myogenic factors, consistent with the in vivo observations (Figure 3E). These results at the cellular level confirmed that diosgenin acts on myogenic lineage cells, regulating key myogenic factors to drive their differentiation and maturation.

### 3.4 Diosgenin Upregulated the Transcription and Translation of Myokines METRNL and IGF‐1

**FIGURE 4:**
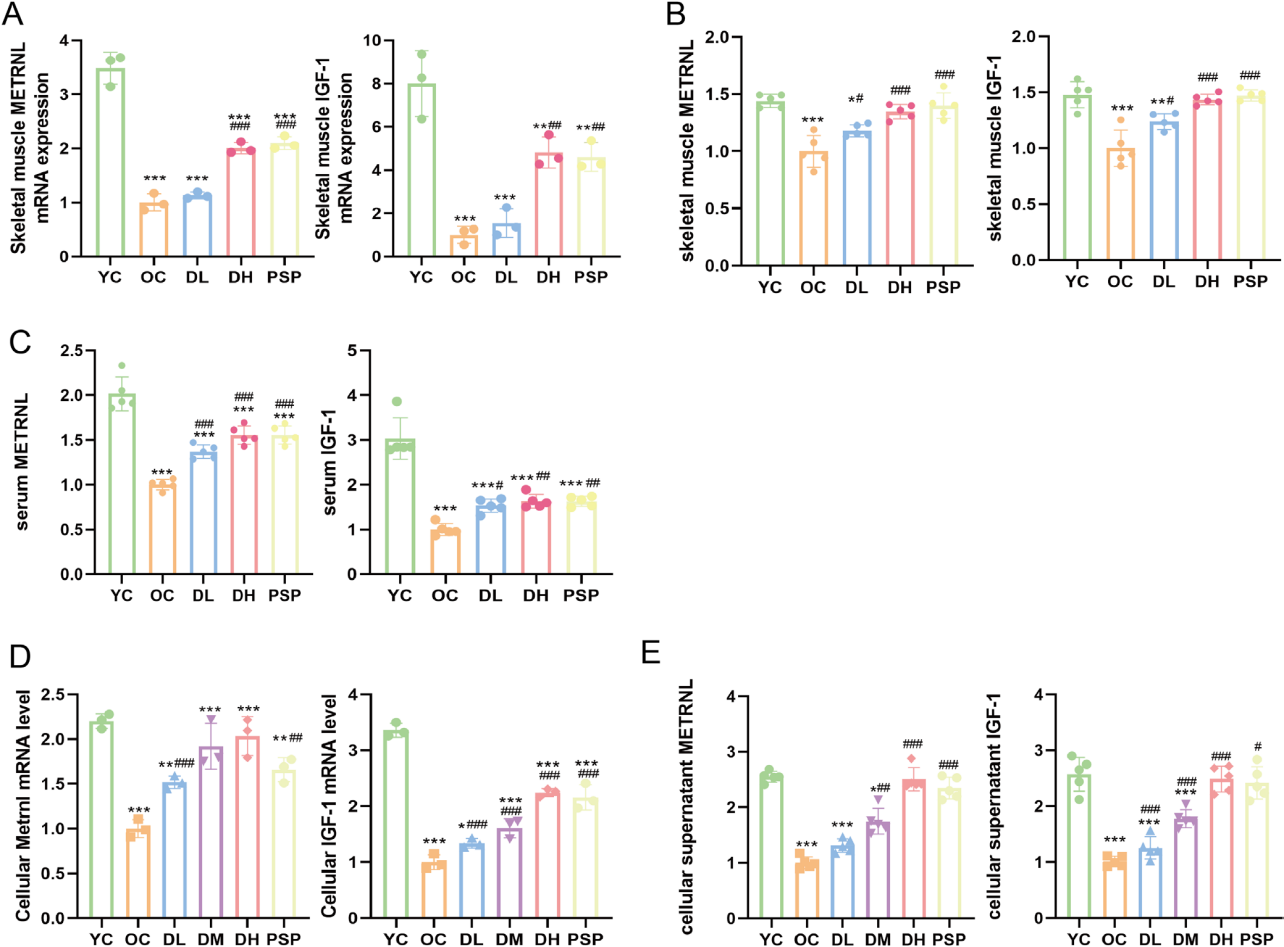
Diosgenin upregulated the transcription and translation of myokines METRNL and IGF‐1. (A) mRNA expression levels of *Metrnl* and *Igf1* in mouse gastrocnemius muscle tissues. (B) Protein concentrations of METRNL and IGF‐1 in mouse gastrocnemius muscle homogenates. (C) Protein concentrations of METRNL and IGF‐1 in mouse serum. (D) mRNA expression levels of *Metrnl* and *Igf1* in C2C12 cells. (E) Protein concentrations of METRNL and IGF‐1 in the culture supernatants of C2C12 cells. Data are presented as mean ± SEM. *p‐value < 0.05, **p‐value < 0.01, ***p‐value < 0.001 versus the YC group; ^#^ *p*‐value < 0.05, ^##^ *p*‐value < 0.01, ^###^ *p*‐value < 0.001 versus the OC group. Statistical analysis was performed using one‐way ANOVA followed by Tukey's multiple comparisons test. All experiments were performed with at least three independent replicates.

As critical signaling molecules, myokines play a pivotal role in the microenvironment that supports satellite cell function recovery. METRNL is a novel exercise‐mimetic factor secreted by skeletal muscle and adipose tissue, while IGF‐1 is a core factor promoting muscle growth and repair. qPCR results showed that, compared with the YC group, the mRNA levels of METRNL and IGF‐1 in the gastrocnemius muscle of the OC group were significantly decreased (*p*‐value < 0.05). ELISA analysis indicated that protein concentrations of METRNL and IGF‐1 in both gastrocnemius muscle homogenates and serum were also significantly reduced in the OC group (*p*‐value < 0.05). Following intervention, both the DH and positive control PSP groups exhibited significant, comparable increases in METRNL and IGF‐1 at both the mRNA and protein levels (*p*‐value < 0.05). The DL group also showed significant increases at the protein level, though not at the mRNA level (Figure 4A–C).

In the C2C12 cell model, both diosgenin (in a dose‐dependent manner) and PSP treatment significantly increased the mRNA levels of METRNL and IGF‐1, along with their protein levels in the culture supernatant, consistent with the in vivo observations (Figure 4D,E). These results demonstrated that diosgenin upregulated key myokines METRNL and IGF‐1 at both transcriptional and translational levels, which may represent an important mechanism by which it alleviates age‐related sarcopenia.

### 3.5 Diosgenin Alleviated Age‐Related Sarcopenia by Activating the SIRT1/PGC‐1α Signaling Pathway

**FIGURE 5:**
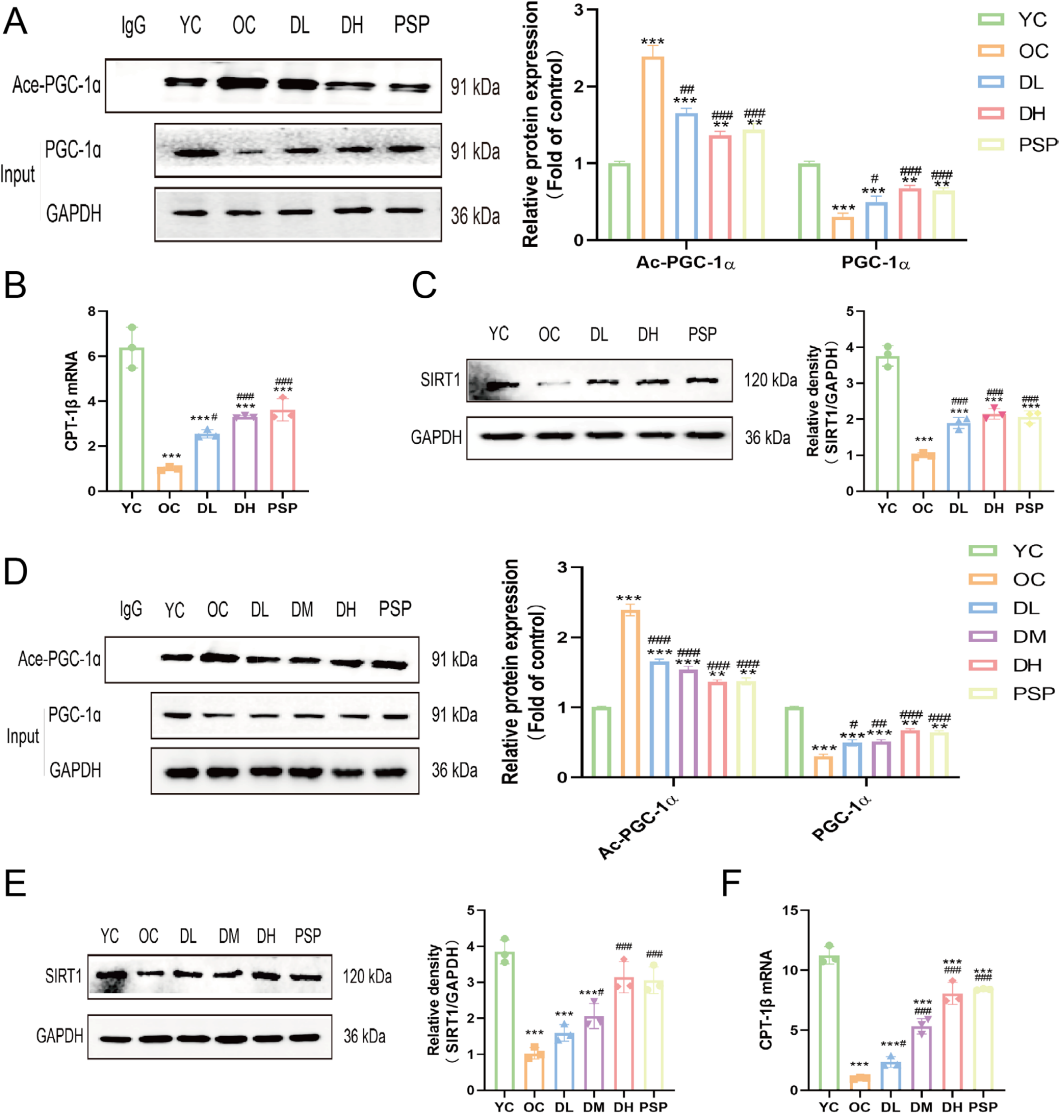
Diosgenin activated the SIRT1/PGC‐1α signaling pathway. (A) Representative WB images of PGC‐1α IP and acetylation and quantitative analysis in mouse skeletal muscle. (B) mRNA expression levels of the mitochondrial function–related gene *Cpt1b* in mouse gastrocnemius muscle. (C) Representative WB images and quantitative analysis of SIRT1 in mouse skeletal muscle. (D) Representative images and quantitative analysis of PGC‐1α immunoprecipitation and acetylation in C2C12 cells. (E) mRNA expression levels of *Cpt1b* in C2C12 cells. (F) Representative WB images and quantitative analysis of SIRT1 in C2C12 cells. Data are presented as mean ± SEM. **p‐value < 0.01, ***p‐value < 0.001 versus the YC group; ^#^ *p*‐value < 0.05, ^##^ *p*‐value < 0.01, ^###^ *p*‐value < 0.001 versus the OC group. Statistical analysis was performed using one‐way ANOVA followed by Tukey's multiple comparisons test. All experiments were performed with at least three independent replicates.

To clarify the core molecular mechanism of diosgenin, we systematically examined SIRT1/PGC‐1α–related changes, the upstream regulatory axis of METRNL and IGF‐1. As a key regulator of METRNL expression in muscle, PGC‐1α activity is finely modulated by acetylation. IP assays showed that total PGC‐1α protein expression was decreased in the OC group, while acetylation levels were significantly increased (*p*‐value < 0.05), resulting in impaired transcriptional coactivator function. Diosgenin treatment markedly increased PGC‐1α protein expression and enhanced its deacetylation (*p*‐value < 0.05). Notably, the DH group exhibited the most pronounced effect, even slightly outperforming the positive control PSP (Figure 5A). Concurrently, mRNA levels of the PGC‐1α downstream mitochondrial function‐related gene *Cpt1b* were significantly upregulated by both diosgenin and PSP, reflecting effective regulation of PGC‐1α (Figure 5B). To further investigate the molecular basis for enhanced PGC‐1α deacetylation, we assessed the expression of its upstream deacetylase, SIRT1. WB results revealed that SIRT1 protein expression in the skeletal muscle of OC mice was significantly decreased (*p*‐value < 0.05). Diosgenin treatment increased SIRT1 expression (*p*‐value < 0.05), with the DH group achieving the most substantial upregulation, reaching levels close to the YC group and exceeding those of the PSP group (Figure 5C). These findings indicated that diosgenin may improve age‐related sarcopenia by targeting the SIRT1/PGC‐1α signaling axis.

Similar changes were observed in the D‐gal–induced C2C12 senescence model. IP and WB analyses confirmed that both diosgenin (in a dose‐dependent manner) and PSP treatment significantly upregulated PGC‐1α and SIRT1 protein expression while reducing PGC‐1α acetylation levels (*p*‐value < 0.05), consistent with the in vivo results (Figure 5D,E). Moreover, qPCR results showed that both interventions markedly increased the mRNA levels of *Cpt1b* (*p*‐value < 0.05) (Figure 5F). These results demonstrated that diosgenin regulated the SIRT1/PGC‐1α signaling axis, suggesting that it played a central role in ameliorating age‐related sarcopenia.

### 3.6 Diosgenin Ameliorated Age‐Related Sarcopenia by Targeting SIRT1

**FIGURE 6:**
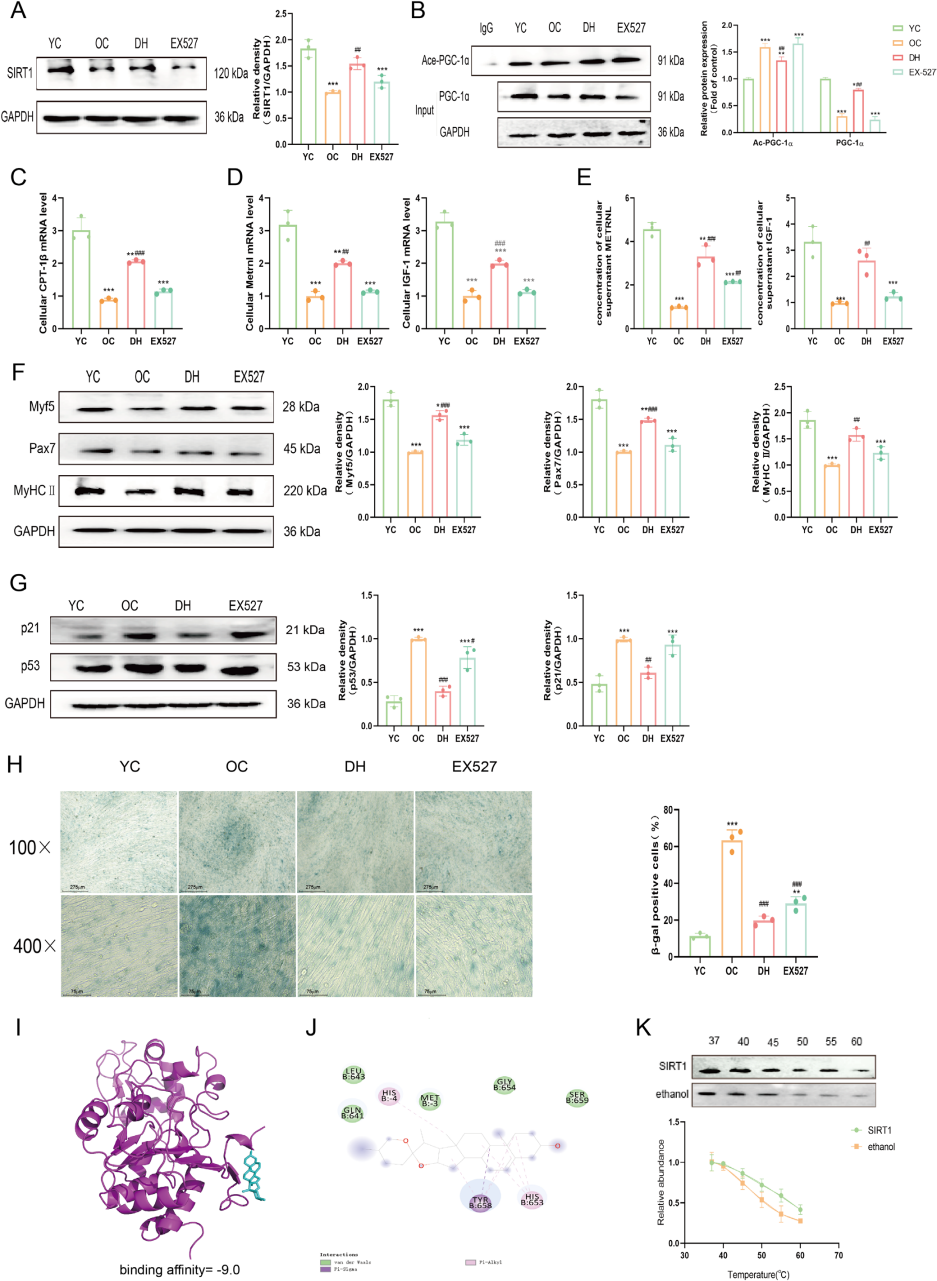
Diosgenin regulated SIRT1 to ameliorate age‐related sarcopenia. (A) Representative WB images and quantitative analysis of SIRT1 in C2C12 cells. (B) Quantitative analysis of PGC‐1α protein expression and PGC‐1α acetylation levels in C2C12 cells. (C) mRNA levels of *Cpt1b* in C2C12 cells. (D) mRNA levels of *Metrnl* and *Igf1* in C2C12 cells. (E) Protein concentrations of METRNL and IGF‐1 in C2C12 cells. (F) Representative WB images and quantitative analysis of the myogenic differentiation markers Myf5, Pax7, and MyHC II in C2C12 cells. (G) Representative WB images and quantitative analysis of the senescence markers p53 and p21 in C2C12 cells. (H) Representative SA‐β‐gal staining images of C2C12 cells at 100× and 400× magnification, with quantitative analysis. (I) Molecular docking results of diosgenin with the SIRT1 protein. (J) Schematic diagram of the interactions between diosgenin and key residues of SIRT1. (K) Representative WB images reflecting the thermal stability of SIRT1 protein under different temperature gradients and the corresponding thermal stability curve; the y‐axis represents the remaining SIRT1 protein level relative to 37°C. Data are presented as mean ± SEM. *p‐value < 0.05, **p‐value < 0.01, ***p‐value < 0.001 versus the YC group; ^#^ *p*‐value < 0.05, ^##^ *p*‐value < 0.01, ^###^ *p*‐value < 0.001 versus the OC group. Statistical analysis was performed using one‐way ANOVA followed by Tukey's multiple comparisons test. All experiments were performed with at least three independent replicates.

To confirm the target of diosgenin, the SIRT1‐specific inhibitor EX527 was applied in the D‐gal‐induced C2C12 cell senescence model. The results showed that EX527 significantly suppressed the upregulation of SIRT1 protein induced by diosgenin. Meanwhile, the promotive effect of diosgenin on PGC‐1α deacetylation was blocked, resulting in an increase in PGC‐1α acetylation level (*p*‐value < 0.05), and the upregulation of PGC‐1α protein expression by diosgenin was abolished (*p*‐value < 0.05) (Figure 6A,B). Under EX527 treatment, the improvement of the PGC‐1α downstream regulator CPT‐1β by diosgenin was also markedly inhibited (*p*‐value < 0.05) (Figure 6C). Further analysis revealed that SIRT1 inhibition significantly suppressed diosgenin‐induced upregulation of the myokines METRNL and IGF‐1 at both mRNA and protein levels (*p*‐value < 0.05) (Figure 6D,E). Similarly, the enhancing effects of diosgenin on the expression of myogenic differentiation markers Myf5, Pax7, and MyHC II were reversed by EX527 (*p*‐value < 0.05) (Figure 6F). In addition, the inhibitory effects of diosgenin on senescence markers p53 and p21 were partially or completely reversed (*p*‐value < 0.05), and the proportion of SA‐β‐gal‐positive cells increased again (Figure 6G,H). These results indicated that the ameliorative effects of diosgenin on age‐related sarcopenia depended on the SIRT1 pathway.

Molecular docking analysis revealed that diosgenin formed multiple stable interactions with SIRT1: van der Waals interactions at residues LEU643 and GLN641, and hydrophobic interactions including Pi‐Alkyl and Pi‐Sigma at HIS B:‐4, HIS B:653, and TYR B:658. The binding energy was −9.0 kcal/mol, indicating a stable binding affinity between diosgenin and SIRT1 (Figure 6I,J). Furthermore, CETSA experiments confirmed the direct interaction between diosgenin and SIRT1. Compared with the solvent control, diosgenin‐treated cells retained more SIRT1 protein at higher temperatures, and the thermal denaturation curve shifted to the right, indicating enhanced thermal stability of SIRT1 upon diosgenin binding (Figure 6K).

Collectively, these results demonstrated that diosgenin targeted SIRT1, deacetylation‐activated PGC‐1α signaling, upregulated myokine expression, enhanced satellite cell proliferation and differentiation, and ultimately improved age‐related sarcopenia.

## 4 Discussion

Aging is characterized by a progressive decline in tissue regenerative capacity, with skeletal muscle being particularly susceptible to age‐related functional deterioration (Mao et al. 2025). In this study, the effects of diosgenin on age‐related sarcopenia were systematically evaluated, and its molecular mechanism was elucidated: diosgenin targeted SIRT1, mediated the expression and deacetylation activation of PGC‐1α, thereby regulating myokine expression as well as satellite cell proliferation and myogenic differentiation, ultimately improving age‐related declines in skeletal muscle function and mass. These findings not only revealed the core mechanism by which diosgenin counteracts age‐related sarcopenia, but also provided new pharmacological evidence supporting the SIRT1/PGC‐1α signaling axis as a therapeutic target for sarcopenia.

Diosgenin, the key active component of *P. sibiricum*, has been used in traditional Chinese medicine for centuries and possesses the safety advantages of a natural product (Mandal et al. 2025). Modern pharmacological studies have demonstrated that diosgenin exerts anti‐aging and metabolic regulatory effects in various tissues (El‐Far et al. 2024; Kanchan et al. 2016; Liu, Shen, et al. 2024; Nie et al. 2023). Previous studies suggested that crude extracts of *P. sibiricum* could improve exercise endurance and resist fatigue, but systematic investigations on the effects of its main active component, diosgenin, in age‐related sarcopenia are still lacking (Zhao et al. 2018).

The essence of age‐related sarcopenia is the degenerative change of skeletal muscle, manifested as reduced muscle strength, decreased endurance, and muscle fiber atrophy (Duan et al. 2025; Rospleszcz et al. 2025). In this study, the ameliorative effects of diosgenin were first validated at the animal level. After 8 weeks of diosgenin intervention, aged mice exhibited increased forelimb grip strength, prolonged weighted swimming time, elevated gastrocnemius index, and enlarged muscle fiber cross‐sectional area. This study also confirmed that diosgenin could reverse muscle fiber atrophy, restoring the cross‐sectional area distribution toward a normal pattern. Furthermore, diosgenin significantly reduced the expression of senescence markers in skeletal muscle, including p16, p53, and p21, indicating its ability to delay aging at the cellular level. These findings provide important in vivo pharmacodynamic evidence for diosgenin as a candidate natural product against age‐related sarcopenia and lay a foundation for subsequent clinical translation studies.

Satellite cell dysfunction is a core mechanism of age‐related sarcopenia, and the restoration of their proliferative and differentiation capacity is crucial for reversing declines in muscle mass and function (Bachman and Chakkalakal 2024). During aging, satellite cells not only decrease in number, but their microenvironment also undergoes significant alterations, including reduced muscle factor levels and changes in the extracellular matrix, collectively impairing self‐renewal and differentiation capacity (Kamal et al. 2025; Qiu et al. 2025; Riparini et al. 2025). In this study, diosgenin was shown to improve satellite cell function at multiple levels. First, Pax7, a marker of satellite cells, is essential for maintaining their stemness and proliferative potential (Seale et al. 2000). Addicks et al. reported that the decline of Pax7 expression during aging is closely associated with impaired proliferation and differentiation, whereas maintaining Pax7 expression can delay satellite cell senescence (Addicks et al. 2019). Our results demonstrated that diosgenin treatment increased the total number of Pax7^+^ satellite cells and significantly elevated the proportion of Pax7^+^/Ki67^+^ proliferative cells, indicating that satellite cells were activated from quiescence and entered the proliferative phase, providing a molecular basis for the anti‐senescence effect of diosgenin on satellite cells. Second, myogenic differentiation is a key step for satellite cells to execute their repair function (Bronisz‐Budzynska et al. 2022). Myf5, a key member of the myogenic regulatory factor family, primarily governs early cell fate and its activation marks the initiation of differentiation, while MyHC II, a structural protein of mature muscle fibers, is upregulated during differentiation (Cai et al. 2020; Yokoyama et al. 2025). In this study, diosgenin upregulated the expression of Myf5, Pax7, and MyHC II, thereby activating the myogenic transcriptional network and promoting satellite cell differentiation toward mature myocytes. Notably, excessive proliferation can exhaust the stem cell pool, while promoting differentiation alone does not expand cell numbers (Zhang et al. 2023). We observed that the total number of Pax7^+^ cells increased concurrently with the upregulation of differentiation markers Myf5 and MyHC II, suggesting that diosgenin coordinated the “proliferation–differentiation” balance of satellite cells, which is critical for the long‐term maintenance of muscle regenerative capacity.

Satellite cell proliferation and differentiation are tightly regulated by signaling molecules within the microenvironment, among which myokines play a pivotal role (Guo, Yao, et al. 2023; Wang et al. 2025). Myokines are bioactive proteins secreted by skeletal muscle, including METRNL and IGF‐1 (Zhou et al. 2025). METRNL, recognized as an exercise‐mimetic factor, acts on satellite cells through autocrine and paracrine mechanisms, promoting their transition from a quiescent to an activated state and contributing to exercise adaptation and muscle regeneration. Previous studies reported that METRNL enhanced the proliferation of Pax7^+^ satellite cells while maintaining their stem cell properties (Baht et al. 2020). In the present study, diosgenin treatment increased METRNL levels in both serum and skeletal muscle homogenates, thereby establishing a microenvironment favorable for satellite cell activation. IGF‐1 is another critical promoter of muscle growth, and attenuation of IGF‐1 signaling during aging is considered a major contributor to the development of sarcopenia (Baht et al. 2020). Activation of IGF‐1R and its downstream signaling cascades has been shown to stimulate satellite cell proliferation and drive myogenic differentiation, while IGF‐1 overexpression markedly increases muscle mass and strength in aged mice (Ungvari and Csiszar 2012). Our results demonstrated that diosgenin restored IGF‐1 levels in the serum and skeletal muscle of aged mice, which may represent an important mechanism underlying its pro‐myogenic effects. Collectively, these findings suggested that diosgenin promoted a regenerative microenvironment by upregulating the expression and secretion of the myokines METRNL and IGF‐1, thereby facilitating satellite cell proliferation and differentiation and ultimately enhancing skeletal muscle regeneration under aging conditions.

In the present study, we further investigated the key mechanisms underlying the protective effects of diosgenin against age‐related sarcopenia. PGC‐1α is a central transcriptional regulator of the METRNL and IGF‐1 gene promoters. Previous studies demonstrated that increased intramuscular PGC‐1α induced transcriptional activation of METRNL, while METRNL in turn stimulated IGF‐1 secretion via a signal transducer and activator of transcription 3 (STAT3)‐dependent pathway (Guo, Zhang, et al. 2023; Liu, Wang, et al. 2024). As a transcriptional coactivator, the functional activity of PGC‐1α is tightly regulated by post‐translational acetylation. During aging, PGC‐1α protein expression declines, accompanied by increased acetylation and impaired transcriptional coactivator activity, which was consistent with the alterations observed in our aged animal and cellular models. Following diosgenin treatment, PGC‐1α expression was markedly increased, accompanied by enhanced deacetylation and functional activation. Concurrently, both mRNA and protein levels of METRNL and IGF‐1 were significantly upregulated, suggesting that activation of PGC‐1α may represent a key mechanism by which diosgenin ameliorated sarcopenia. Moreover, CPT‐1β, a downstream target of PGC‐1α and the rate‐limiting enzyme for mitochondrial long‐chain fatty acid β‐oxidation, was also upregulated in parallel with enhanced PGC‐1α signaling. This finding further supported a positive regulatory effect of diosgenin on the PGC‐1α pathway and its associated mitochondrial metabolic functions.

The abundance and activity of PGC‐1α are precisely regulated by upstream signaling pathways, among which SIRT1 is a critical regulatory factor. On the one hand, SIRT1 upregulates PGC‐1α protein expression; on the other hand, SIRT1 enhances the transcriptional coactivator activity of PGC‐1α by deacetylating multiple lysine residues. Consequently, the SIRT1/PGC‐1α axis plays a central role in the regulation of skeletal muscle function and myocyte regeneration. In the present study, SIRT1 protein expression was significantly reduced in the skeletal muscle of aged mice, accompanied by impaired deacetylation‐dependent activation of PGC‐1α. Following diosgenin intervention, SIRT1 expression was restored. However, when the SIRT1‐specific inhibitor EX527 was applied, the diosgenin‐induced upregulation of SIRT1 was abolished, leading to reduced PGC‐1α expression and blockade of its deacetylation‐mediated activation. Accordingly, the diosgenin‐induced increases in METRNL and IGF‐1 expression at both mRNA and protein levels were completely suppressed. In parallel, the improvements in satellite cell proliferation and differentiation markers were eliminated, and the inhibitory effects on senescence‐associated markers were reversed. These findings indicated that SIRT1 was a critical molecular target mediating the protective effects of diosgenin against age‐related sarcopenia. To further substantiate this hypothesis, molecular docking analysis demonstrated that diosgenin formed a stable binding conformation within the active pocket of SIRT1. In addition, CETSA experiments confirmed a direct physical interaction between diosgenin and SIRT1. Collectively, these results demonstrated that diosgenin directly targeted SIRT1, activated the SIRT1/PGC‐1α signaling pathway, and ultimately ameliorated age‐related sarcopenia.

Nevertheless, several limitations of the present study should be acknowledged. First, although pharmacological inhibition, molecular docking, and CETSA analyses collectively supported a direct interaction between diosgenin and SIRT1, the necessity and sufficiency of SIRT1 in mediating the muscle‐protective effects of diosgenin were not rigorously established. Future studies employing muscle‐specific SIRT1 knockout mouse models would provide more definitive genetic evidence. Second, the present work primarily focused on the SIRT1/PGC‐1α/METRNL axis; however, PGC‐1α regulates a broad spectrum of downstream target genes. Future investigations integrating transcriptomic and acetyl‐proteomic analyses would be valuable for delineating a more comprehensive regulatory network underlying the effects of diosgenin.

## 5 Conclusions

Overall, the present study demonstrated that diosgenin directly activated the SIRT1/PGC‐1α signaling pathway, upregulated the expression of the myokines METRNL and IGF‐1, and promoted satellite cell proliferation and myogenic differentiation, thereby ameliorating age‐related declines in skeletal muscle mass and function. These findings suggest that diosgenin may represent a promising candidate for the prevention and treatment of age‐related sarcopenia and may provide a novel therapeutic strategy to support healthy aging.

## Author Contributions

**Xin Zeng:** methodology, investigation, validation, visualization, writing – original draft. **Han Ding:** methodology, software, validation, formal analysis, data curation, visualization, writing – original draft. **Ziye Li:** investigation, resources. **Qian Wang:** investigation. **Peiyao Guan:** validation. **Tingting Wang:** investigation. **Jiayun Wang:** formal analysis. **Yansong Fu:** investigation. **Lizhang Chen:** conceptualization, writing – review, supervision, project administration. **Hong Qin:** conceptualization, writing – review and editing, supervision, project administration, funding acquisition.

## Funding

This research was funded by the National Natural Science Foundation of China (No. 82574088) and the Natural Science Foundation of Hunan Province (No. 2025JJ50654).

## Ethics Statement

The experimental protocol was approved by the Animal Welfare and Ethics Committee of Central South University (Approval number: CSU‐2024‐0182).

## Conflicts of Interest

The authors declare no conflicts of interest.

## Data Availability

The data supporting the findings of this study are available from the corresponding author upon reasonable request.
